# Erythema Nodosum

**Published:** 1907-10-05

**Authors:** 


### Diseases of Children.

### ERYTHEMA NODOSUM.

It is illustrative of the difficulties in the complete understanding of disease that the etiology of an apparently simple affection, such as erythema nodosum, is capable of raising a stormy discussion. This is a problem which the family practitioner has much better chances of solving than the physician or skin specialist. Not only does he see the patient throughout the illness, but he is likely to know the family and past history of the child, and to be able to follow its subsequent health and career.

### Theories of Causation.

According to one view the disease is due to rheumatism; to another, it has nothing to do with the rheumatic poison, and is allied to urticarial affections. It has been named "nodal fever," and regarded as an acute specific febrile disorder with an incubation period, prodromata, a stage of eruption, and a period of convalescence. No organism has been isolated, though its course and symptomatology suggest microbial infection.

Many attacks are ushered in by gastro-intestinal disturbance; some are preceded by tonsillitis. Perhaps the eruption is due to absorption of toxins from the alimentary tract or the tonsils. It occurs in its typical form in children, but is rare under three years of age. About 20 per cent. of the cases occur in the first decade, 40 per cent. in the second, and 20 per cent. in the third decade of life. It is much more frequent in females than males, the relative preponderance of females increasing as age advances, the reverse of what holds good for rheumatic fever.

### The Rheumatic Theory.

In favour of the rheumatic theory may be mentioned the occurrence of erythema and the liability to arthritis, tonsillitis, and endocarditis. Both diseases may affect more than one in a family, and may assume a house or epidemic form. Against it are the facts that the disease is least prevalent in the third quarter of the year, when rheumatic fever is most common; that the arthritis may not be of rheumatic character; that the disease is uninfluenced by salicylates.

Lenden states that there was no family history of a rheumatic nature in 63 cases in 49 families, and only two had a past history of rheumatic fever. On the other hand, I obtained such a history in 9 out of 24, but a personal history in only one. According to Goodhart and Still, 19 out of 29 cases were rheumatic, whereas J. Odery Symes states that rheumatic signs are not present in more than 10 per cent. of all cases. Arthritic symptoms are uncommon or slight. They take the form of pain in the joints, with little tenderness on movement, but no redness or swelling such as is seen in the acute rheumatism of adults; yet they are comparable to the rheumatic joint affection of early life. Sometimes the swelling of the joints may simulate gout, gonorrhœal rheumatism, or osteo-arthritis. Mackenzie found arthritis in 15.7 per cent. of 108 cases (males 18.0, females 9.0), and rheumatic pains in another 15.7 per cent., while no less than 21.3 per cent. had or developed signs of endocarditis. Too much importance must not be attached to such figures, for the differential diagnosis of the murmur of cardiac dilatation from that of endocarditis depends largely on the personal equation. A child may be ailing, "headachy," dyspeptic, or feverish, for one to four weeks before the outbreak. Sometimes there is tonsillitis or an attack of diarrhœa. The onset may be gradual or acute, with vomiting or gastro-enteritis and fever. The temperature rarely rises above 104° F., and usually subsides by lysis in the second week. The nodes appear in one to five days, and are almost always exquisitely tender and painful; bright or dusky red to purple in colour; chiefly on the shins, less often on knees, thighs, forearm, and buttocks. Sometimes the rash is polymorphic. The constitutional symptoms are rarely severe, and do not vary with the extent of the rash. There may be much prostration and mental depression. Sometimes delirium and coma are due to hyperpyrexia or to salicylates. Œdema of the legs may be marked. The purple colour is most striking when the limbs are cold and the rash fading.

### Course and Progress.

Convalescence is slow. There are weakness and anæmia out of proportion to the severity of the attack. Relapses are infrequent, and recurrence is rare. Phlyctenulæ are not uncommon. Other complications are angina, bronchitis, arthritis, endocarditis, pleurisy, and nephritis. Certainly cardiac affections seem more prevalent among these children, but neither a febrile arthritis nor endocarditis is proof of acute rheumatism.

### Diagnosis and Treatment.

The diagnosis is easy when the rash is out. Before that the fever may be ascribed to influenza, gastritis, rheumatism, typhoid fever, or even meningitis, if the constitutional symptoms are severe. The prognosis is excellent, though it may prove serious if cardiac mischief should develop, yet I have never known a case turn out badly. The important point in treatment is to put the child to bed until the nodes have subsided and there is no fear of secondary swelling of the legs. Local applications of lead, or lead and opium lotion, relieve the pain. Occasionally hot fomentations are more soothing. The bowels should be opened by a hydragogue purge. The diet must be restricted at first to milk and carbohydrate foods. Oranges and lemons can be given freely. Citrates, citric acid, and quinine are useful drugs. The heart must be watched. Special care is necessary for a few weeks during convalescence, with the exhibition of iron tonics or cod-liver oil.

